# QTL Analysis of Na^+^ and K^+^ Concentrations in Roots and Shoots under Different Levels of NaCl Stress in Rice (*Oryza sativa* L.)

**DOI:** 10.1371/journal.pone.0051202

**Published:** 2012-12-06

**Authors:** Zhoufei Wang, Zhiwei Chen, Jinping Cheng, Yanyan Lai, Jianfei Wang, Yongmei Bao, Ji Huang, Hongsheng Zhang

**Affiliations:** The Laboratory of Seed Science and Technology, State Key Laboratory of Crop Genetics and Germplasm Enhancement, Nanjing Agricultural University, Nanjing, People’s Republic of China; Kansas State University, United States of America

## Abstract

The key to plant survival under NaCl salt stress is maintaining a low Na^+^ level or Na^+^/K^+^ ratio in the cells. A population of recombinant inbred lines (RILs, F_2∶9_) derived from a cross between the salt-tolerant *japonica* rice variety Jiucaiqing and the salt-sensitive *indica* variety IR26, was used to determine Na^+^ and K^+^ concentrations in the roots and shoots under three different NaCl stress conditions (0, 100 and 120 mM NaCl). A total of nine additive QTLs were identified by QTL Cartographer program using single-environment phenotypic values, whereas eight additive QTLs were identified by QTL IciMapping program. Among these additive QTLs, five were identified by both programs. Epistatic QTLs and QTL-by-environment interactions were detected by QTLNetwork program in the joint analyses of multi-environment phenotypic values, and one additive QTL and nine epistatic QTLs were identified. There were three epistatic QTLs identified for Na^+^ in roots (RNC), three additive QTLs and two epistatic QTLs identified for Na^+^ in shoots (SNC), four additive QTLs identified for K^+^ in roots (RKC), four additive QTLs and three epistatic QTLs identified for K^+^ in shoots (SKC) and one additive QTL and one epistatic QTL for salt tolerance rating (STR). The phenotypic variation explained by each additive, epistatic QTL and QTL×environment interaction ranged from 8.5 to 18.9%, 0.5 to 5.3% and 0.7 to 7.5%, respectively. By comparing the chromosomal positions of these additive QTLs with those previously identified, five additive QTLs, *qSNC9*, *qSKC1*, *qSKC9*, *qRKC4* and *qSTR7*, might represent novel salt tolerance loci. The identification of salt tolerance in selected RILs showed that a major QTL *qSNC11* played a significant role in rice salt tolerance, and could be used to improve salt tolerance of commercial rice varieties with marker-assisted selection (MAS) approach.

## Introduction

Salt stress is one of the most important abiotic stresses affecting crop productivity worldwide. For plants, sodium ions (Na^+^) are harmful, whereas potassium ions (K^+^) are essential to reduce the uptake of Na^+^ [1]–[2]. Several studies support the idea that many transporters, such as members of HAK/KUP/KT, HKT and LCT families, play important roles in the uptake and translocation of K^+^ and Na^+^ in plants [2]–[5]. The *SKC1* QTL in rice, which has a major effect on K^+^ concentration, was cloned and identified as the sodium transporter *OsHTK8* [6]. Because rice salt tolerance is a complex quantitative trait and it is difficult to elucidate the genetic control of salt tolerance through the study of a single gene, the genetic basis of salt tolerance in rice is widely documented through QTL analysis, comparative genomic and transcriptome analysis [7]–[9].

In rice, QTL analysis of Na^+^ uptake, K^+^ uptake and ion balance is used for genetic analysis and also to facilitate plant breeding using marker-assisted selection (MAS). A large number of QTLs involved in Na^+^ and K^+^ concentrations of roots and shoots have been identified [10]–[16]. For example, ten QTLs for salt tolerance parameters, including Na^+^ and K^+^ uptake, Na^+^ and K^+^ concentrations and Na^+^/K^+^ ratio in shoots were identified by Koyama et al. [10]. Bonilla et al. [11] mapped the *SALTOL* locus, which is linked to QTLs for Na^+^ and K^+^ uptake and Na^+^/K^+^ ratio, on chromosome 1. Lin et al. [12] mapped QTLs for root and shoot Na^+^/K^+^ concentrations and transport on twelve rice chromosomes. Ammar et al. [13] reported 25 QTLs for salt ion concentrations on rice chromosomes 1, 2, 3 and 8. Pandit et al. [14] reported eight QTLs for salt ion concentrations on rice chromosomes 1, 8 and 12, and Cheng et al. [16] reported twelve QTLs for salt ion concentrations on rice chromosomes 1, 2, 3, 4, 7 and 11. These studies focused on the additive QTLs for Na^+^ and K^+^ and Na^+^/K^+^ ratios in roots and shoots under a single level of NaCl stress; in contrast, few studies have reported epistatic QTL and QTL×environment interaction analyses under different levels of NaCl stress [16].

The complex epistatic and interaction effects between QTLs and environment are important in controlling the quantitative traits [17]–[20]. As rice salt tolerance is a complex trait, in addition to the additive QTLs, it is necessary to identify epistatic QTLs and the interaction of QTLs and environment in rice for salt tolerance. It is difficult to correlate the epistatic QTLs and QTL×environment interactions with the pattern of epistasis, and the interaction with environment is very complex for some traits [21]. Some programs, such as QTLNetwork, genotype matrix mapping (GMM), QTL Mapper, IciMapping program and the multiple interval mapping (MIM) of QTL Cartographer, have been used to identify epistatic QTLs in several crop species [16], [22]–[26], and QTLNetwork and GMM have also been used to identify the interaction of QTLs with environment [19], [24].

Although several QTLs for salt tolerance have been identified in rice, the comparison of additive QTLs for Na^+^ and K^+^ concentrations in roots and shoots under different salt stress conditions has rarely been conducted, and the epistasis and interaction between QTLs and environment for rice salt tolerance remains unknown. In this study, a RIL population derived from a cross between *japonica* Jiucaiqing (salt tolerant) and *indica* IR26 (salt sensitive) [25] was used. We examined the additive QTLs for Na^+^ and K^+^ concentrations in roots and shoots under different levels of NaCl stress (0, 100 and 120 mM NaCl) using Cartographer [27] and IciMapping [28] approaches, and the epistatic QTLs and interaction between QTLs and environment were also analyzed using QTLNetwork approach [29]. These QTLs can be used in improving salt tolerance in rice by MAS.

## Materials and Methods

### Plant Materials

Two rice varieties, Jiucaiqing (*japonica*) and IR26 (*indica*), and their 150 RIL lines (F_2∶9_) were used in this study. In our previous experiments, we found that Jiucaiqing was highly salt tolerant, whereas IR26 was salt sensitive [25].

### Evaluation of Salt Tolerance

A sample of fifty healthy grains from each parent and RIL were surface-sterilized with 0.1% mercuric chloride solution for 10 min and then rinsed three times with sterile distilled water. The seeds were soaked in distilled water at 30°C for 3 days to allow the seed germination. The germinated seeds were sown in a plastic box (40 cm×30 cm×18 cm) filled with 1.5 kg of quartz sand and placed in a growth chamber at 30°C. At the three-leaf seedling stage, the well-established, uniform seedlings were transplanted to 1-cm plugged holes in foam sheets floated over 15 liters of nutrient solution [30] in the plastic box for 7 days. For salt treatment, the nutrient solution was replaced with a fresh solution containing 100 or 120 mM NaCl for 10 days. The culture solution was refreshed every 2 days, and the pH was maintained at 5.6. After 10 days of salt stress, the salt tolerance rating (STR) was assessed from 0 to 5 [31]. Here 0 means no damage; 1 means very slight damage; 2 means 25–50% of leaves yellow; 3 means 50–75% of leaves yellow; 4 means more than 75% of leaves yellow and 5 means all seedlings dead. The Na^+^ and K^+^ in shoots and roots of each sample were extracted using 6.5 mL nitrification liquid [32] including 60% trichloroacetic acid (1 mL), 98% nitric acid (5 mL) and 98% sulfuric acid (0.5 mL), and the concentrations in roots (RNC and RKC) and shoots (SNC and SKC) were analyzed by atomic absorption spectroscopy using a TAS-986 machine (PGENERAL, Beijing, China).

Two selected lines (lines 4 and 8) with their parents were used for a further salt tolerance evaluation under 120 mM NaCl stress. The seedlings were grown under normal conditions (control) for 26 days; for salt treatment, after growth under normal conditions for 14 days, the nutrient solution was replaced with a fresh solution containing 120 mM NaCl for 9 days, with 3 *days* of *recovery* and *growth*. Seedling fresh weight, seedling height and root length of treatments and control were measured. The experimental design consisted of three replications.

### Data Analysis

The experimental data were analyzed using Statistical Analysis System (SAS) software, and the indices of parents were compared with Student’s *t*-test at the 5% and 1% levels of probability. The correlations of indices were computed using PROC CORR by SAS software [33]. The heritability in broad sense (*H^2^*) was calculated and is expressed as the ratio of total genetic variance (*V_G_*) to phenotypic variance (*V_P_*): *H^2^ = V_G_/V_P_*.

### Construction of Linkage Map

The DNA was extracted from rice seedlings using SDS method [34]. PCR was performed using the procedure of Chen et al. [35], and PCR products were then separated on an 8% non-denaturing polyacrylamide gel and visualized using silver staining method described in Sanguinetti et al. [36]. The Mapmaker/EXP 3.0 program was used to construct a complete linkage map [37]. Lastly, a set of 168 SSR markers, covering most of the rice genome at an average interval of 15 cM, was constructed.

### QTL Mapping

Additive QTLs were identified by the method of composite interval mapping (CIM) using WinQTL Cartographer, version 2.5 [27], and the method of inclusive composite interval mapping (ICIM) in QTL IciMapping ver. 3.1 [28] using single-environment phenotypic values. The QTLNetwork program ver. 2.0, based on a mixed linear model [29], was used to identify the epistatic QTLs and QTL×environment interactions for salt tolerance indices in joint analyses of the multi-environment phenotypic values. The LOD thresholds of QTL were determined by a 1,000 permutation test at a 95% confidence level. The proportion of observed phenotypic variance explained by each additive and epistatic QTL and the corresponding additive effects were also estimated. The QTL nomenclature followed the suggestion of McCouch and CGSNL [38].

## Results

### Salt Tolerance Phenotypes

There were significant differences in the STR, RNC, SNC and SKC between two parents under both NaCl stress conditions (100 and 120 mM NaCl), but no significance for the control condition was found (Table 1). In contrast, the salt-tolerant Jiucaiqing had lower STR, RNC, RKC, SNC and SKC values than the sensitive IR26 under both NaCl stress conditions. The Na^+^ and K^+^ concentrations in roots were lower than in shoots under control and 100 mM NaCl conditions, whereas opposite results were observed under 120 mM NaCl condition. There was a continuous frequency distribution and transgressive segregation in these indicators among RIL population under different levels of NaCl. The heritability of STR, RNC, RKC, SNC and SKC were different under the three levels of NaCl stress, ranging from 63.5% to 98.4% (Table 1).

**Table 1:** Phenotypic performance of relevant traits in two parents and RIL population (Jiucaiqing/IR26) under different NaCl conditions.

NaCl treatments(mM)	Traits^a^(mg/g)	Parents^b^		RIL Population^c^						
		Jiucaiqing	IR26	Mean	Max	Min	SD	Skewness	Kurtosis	Heritability (%)
0	RNC(mg/g)	13.1±1.9	16.6±3.1	16.4	30.1	4.2	4.58	0.38	0.85	95.6
	RKC(mg/g)	25.2±3.7	31.8±7.0	31.1	57.4	13.2	7.51	0.85	1.07	88.8
	SNC(mg/g)	14.9±3.7	12.4±3.1	13.6	34.7	6.5	5.69	1.65	2.72	89.8
	SKC(mg/g)	48.9±4.5	49.1±4.5	53.5	71.8	36.5	5.97	−0.66	1.11	97.6
100	STR	2.6±0.2	3.4±0.3*	3.4	5.0	2.3	0.60	0.91	0.27	94.4
	RNC(mg/g)	34.1±5.4	36.8±4.5*	33.7	57.5	18.8	6.24	0.67	1.47	95.6
	RKC(mg/g)	32.4±5.8	32.4±2.3	31.4	51.3	18.6	5.25	0.57	0.85	98.4
	SNC(mg/g)	51.2±5.4	60.0±2.6**	66.6	115.1	38.4	15.15	0.60	0.09	64.6
	SKC(mg/g)	44.6±2.7	56.8±5.5**	62.1	95.7	37.7	10.25	0.26	0.16	63.5
120	STR	3.1±0.3	4.2±0.2*	3.8	5.0	2.2	0.60	−0.11	−0.35	81.9
	RNC(mg/g)	41.4±2.2	47.6±2.3*	52.7	93.3	27.1	10.35	0.78	1.66	98.0
	RKC(mg/g)	26.8±6.1	27.2±4.1	34.7	49.1	20.9	6.191	0.32	−0.51	94.9
	SNC(mg/g)	126.0±5.6	137.3±7.9**	128.3	202.6	65.0	28.50	0.24	−0.52	86.1
	SKC(mg/g)	72.9±6.5	89.7±2.4**	73.2	115.2	43.7	13.09	0.37	0.43	92.8

a RNC: root Na^+^ concentration; RKC: root K^+^ concentration; SNC: shoot Na^+^ concentration; SKC: shoot K^+^ concentration; STR: salt tolerance rating;

b Means ± SD (standard deviation);

* and **indicates significance at the level of 5% and 1%, respectively, according to Student’s *t*-test;

c RILs sample size n = 150, replications r = 3.

### Correlation of Na^+^ and K^+^ in Roots and Shoots

There were significantly positive correlations between the Na^+^ and K^+^ in roots and shoots under NaCl conditions, but no significant correlations were identified under the control condition (Table 2). The correlation of Na^+^ between roots and shoots was significantly positive only under 100 mM NaCl condition and not under the control and 120 mM NaCl conditions. However, the correlation for K^+^ between roots and shoots was significantly positive only under the control condition. The relationship among the STR and Na^+^ and K^+^ concentrations was also examined, and there were highly significant positive correlations among the STR and Na^+^ concentrations in roots and shoots, respectively, under NaCl stress conditions (Table 2).

**Table 2:** Correlation coefficients between different traits under different NaCl conditions.

NaCl treatments(mM)	traits	STR	RNC	RKC	SNC	SKC
0	RNC	−	1			
	RKC	−	−0.197	1		
	SNC	−	−0.197	0.462**	1	
	SKC	−	0.107	−0.065	0.043	1
100	STR	1				
	RNC	0.322**	1			
	RKC	0.011	0.530**	1		
	SNC	0.656**	0.387**	−0.089	1	
	SKC	−0.120	0.001	−0.112	0.421**	1
120	STR	1				
	RNC	0.290**	1			
	RKC	0.039	0.522**	1		
	SNC	0.231**	−0.019	−0.146	1	
	SKC	−0.171	−0.298**	−0.140	0.664**	1

RNC: root Na^+^ concentration; RKC: root K^+^ concentration; SNC: shoot Na^+^ concentration; SKC: shoot K^+^ concentration; STR: salt tolerance rating;

** indicates significance at the level of 1%.

### Additive QTLs for Seedling Salt Tolerance

#### QTLs for Na^+^ concentration

**Figure 1:**
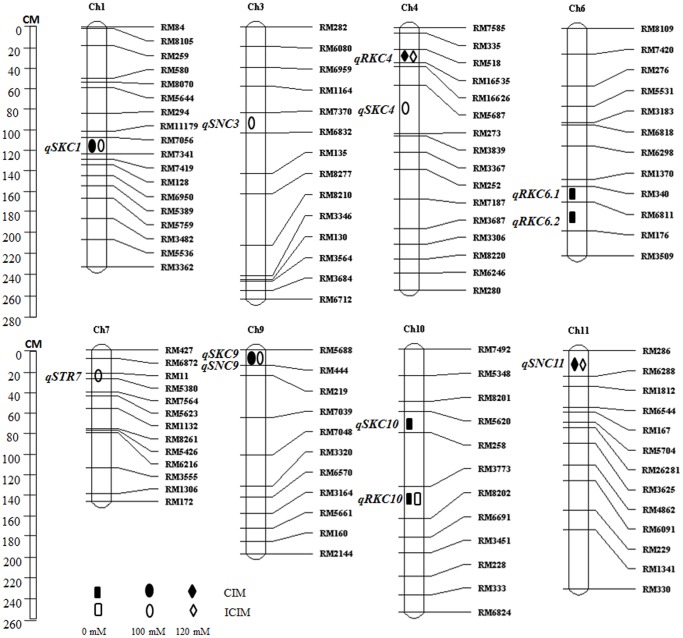
Location of additive QTLs for salt tolerance indices under 0, 100 and 120 mM NaCl conditions on linkage groups by CIM and ICIM.

There were three additive QTLs, *qSNC3*, *qSNC9* and *qSNC11*, mapped for Na^+^ concentration using CIM and ICIM approaches (Table 3; Fig. 1). The QTLs for Na^+^ concentration were detected only in shoots, but not in roots under NaCl stress. Under 100 mM NaCl condition, QTLs *qSNC3* and *qSNC9* were mapped to chromosome 3 and 9 in the marker intervals RM7370-RM6832 and RM5688-RM444, respectively; the LOD scores were 2.5 and 3.3, explaining 13.1 and 8.8% of phenotypic variance, respectively. The positive alleles for both of these QTLs were contributed by Jiucaiqing. Another QTL for Na^+^ in shoot (*qSNC11*) under 120 mM NaCl condition was mapped simultaneously by both CIM and ICIM approaches to chromosome 11 in the marker interval RM286-RM6288; the LOD scores were 4.0 and 3.8, explaining 14.9 and 16.1% of phenotypic variance, respectively. The positive allele for *qSNC11* was contributed by IR26 to increase the SNC.

**Table 3:** Putative additive QTLs for relevant traits in RIL population (Jiucaiqing/IR26) under different NaCl conditions by CIM and ICIM.

Methods	NaCl treatments(mM)	Traits^a^	Chr.^b^	QTLs	Marker interval	LOD	r^2^ (%)^c^	Add^d^
CIM	0	RKC	6	*qRKC6.1*	RM340-RM6811	2.7	9.4	2.5
		RKC	6	*qRKC6.2*	RM6811-RM176	3.2	11.5	2.7
		RKC	10	*qRKC10*	RM3773-RM8202	3.6	9.1	2.4
		SKC	10	*qSKC10*	RM5620-RM258	2.6	12.1	2.2
	100	SNC	9	*qSNC9*	RM5688-RM444	3.3	8.8	4.7
		SKC	1	*qSKC1*	RM7341-RM7419	3.3	9.0	3.3
		SKC	9	*qSKC9*	RM5688-RM444	3.2	10.1	3.3
	120	RKC	4	*qRKC4*	RM518-RM16535	3.3	11.8	2.2
		SNC	11	*qSNC11*	RM286-RM6288	4.0	14.9	−11.4
ICIM	0	RKC	10	*qRKC10*	RM3773-RM8202	3.1	9.1	2.3
	100	STR	7	*qSTR7*	RM11-RM5380	2.8	8.6	0.2
		SNC	3	*qSNC3*	RM7370-RM6832	2.5	13.1	5.5
		SKC	1	*qSKC1*	RM7341-RM7419	4.1	14.3	3.9
		SKC	4	*qSKC4*	RM5687-RM273	2.7	18.9	4.5
		SKC	9	*qSKC9*	RM5688-RM444	2.5	9.2	3.2
	120	RKC	4	*qRKC4*	RM518-RM16535	2.6	8.5	1.8
		SNC	11	*qSNC11*	RM286-RM6288	3.8	16.1	−11.7

a RNC: root Na^+^ concentration; RKC: root K^+^ concentration; SNC: shoot Na^+^ concentration; SKC: shoot K^+^ concentration; STR: salt tolerance rating;

b Chromosome on which the QTL was located;

c Variation explained by each putative QTL;

d Additive effect is the effect of substituting a Jiucaiqing allele for an IR26 allele; Its positive value indicates that Jiucaiqing has the positive allele; The case of negative values is just the opposite.

#### QTLs for K^+^ concentration

There were four additive QTLs, *qRKC6.1*, *qRKC6.2*, *qRKC10* and *qRKC4*, mapped for K^+^ concentration in roots by CIM and ICIM approaches (Table 3; Fig. 1). The QTLs for K^+^ concentration were detected in roots only under control and 120 mM NaCl conditions, whereas no QTLs were detected under 100 mM NaCl condition. The QTLs *qRKC6.1*, *qRKC6.2* and *qRKC10* were mapped under control condition to chromosomes 6 and 10 in the marker intervals RM340-RM6811, RM6811-RM176 and RM3773-RM8202, with LOD scores ranging from 2.7 to 3.6, explaining 9.1 to 11.5% of phenotypic variance. Among them, QTL *qRKC10* was mapped simultaneously by both CIM and ICIM approaches. One QTL for K^+^ in roots (*qRKC4*) under 120 mM NaCl condition was mapped simultaneously by both CIM and ICIM approaches to chromosomes 4 in the marker interval RM518-RM16535, with LOD scores of 3.3 and 2.6, explaining 11.8 and 8.5% of phenotypic variance, respectively. The positive alleles of these QTLs were contributed by Jiucaiqing.

There were four additive QTLs, *qSKC10*, *qSKC1*, *qSKC9* and *qSKC4*, mapped for the K^+^ concentration in shoots by CIM and ICIM approaches (Table 3; Fig. 1). The QTLs for K^+^ concentration were detected only in shoots under control and 100 mM NaCl conditions; no QTL was detected under 120 mM NaCl condition. QTL *qSKC10* was mapped under control condition to chromosome 10 in the marker interval RM5620-RM258, with a LOD score of 2.6, explaining 12.1% of phenotypic variance. Three QTLs, *qSKC1*, *qSKC4* and *qSKC9*, were mapped under 100 mM NaCl condition on chromosomes 1, 4 and 9 in the marker intervals RM7341-RM7419, RM5687-RM273 and RM5688-RM444; the LOD scores ranged from 2.7 to 4.1, explaining 9.0 to 18.9% of phenotypic variance. QTLs *qSKC1* and *qSKC9* were mapped simultaneously by both CIM and ICIM approaches. The positive alleles of these QTLs were contributed by Jiucaiqing.

#### QTLs for STR

There was only one QTL (*qSTR7*) for STR that was mapped under 100 mM NaCl condition by ICIM, and no QTL was detected under 120 mM NaCl condition. The QTL *qSRT7* was located on chromosome 7 in the marker interval RM11-RM5380, with a LOD score of 2.8 that explained 8.6% of phenotypic variance (Table 3; Fig. 1). The positive allele for *qSRT7* was contributed by Jiucaiqing.

### Identification of the QTL×Environment Interactions for Seedling Salt Tolerance

**Figure 2:**
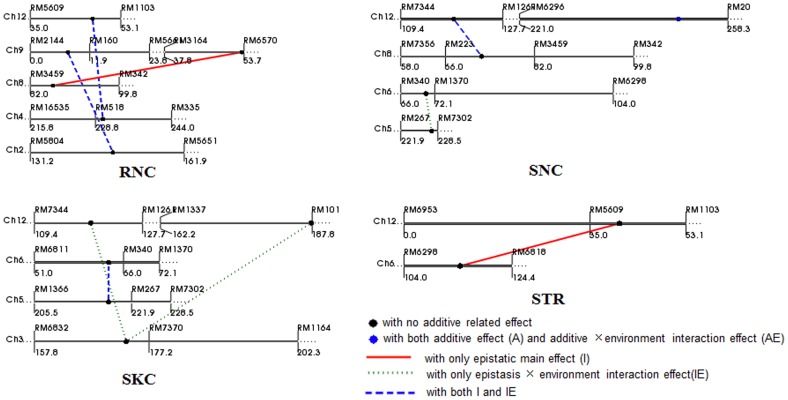
Location of additive and epistatic QTLs for salt tolerance indices with QTL×environment interactions on linkage groups by QTLNetwork.

One additive QTL and nine epistatic QTLs were identified by the combined analysis of the multi-environment phenotypic values under 0, 100 and 120 mM NaCl conditions (Table 4; Fig. 2). Among them, three epistatic QTLs for RNC were detected, two with both an epistatic main effect and epistasis×environment interaction effects and one with only an epistatic main effect. Three QTLs for SNC were detected, one additive QTL with both additive and additive×environment interaction effects, one epistatic QTL with only epistasis×environment interaction effect and one epistatic QTL with both epistatic main effect and epistasis×environment interaction effects. The SKC was controlled by three epistatic QTLs, two with only epistasis×environment interaction effects and one with both an epistatic main effect and an epistasis×environment interaction effect. One epistatic QTL for STR was identified with only an epistatic main effect. The phenotypic variance explained by each QTL ranged from 0.5 to 5.3%, and the phenotypic variation explained by each QTL× environment interaction ranged from 0.7 to 7.5%.

**Table 4:** Epistatic and QE interaction loci for relevant traits of RILs under different NaCl conditions by QTLNetwork.

Traits^a^	loci(*i*)^b^		loci(*j*)^b^		A/AA^c^	AE/AAE^c^			r^2^(A/AA)(%)^d^	r^2^(AE/AAE)(%)^d^
	chr.	intervals	chr.	intervals		AE1/AAE1	AE2/AAE2	AE3/AAE3		
RNC	2	RM5804-RM5651	9	RM2144-RM160	−2.5**	1.6	0.6	−2.2*	4.6	3.0
	4	RM518-RM335	12	RM5609-RM1103	−1.4**	1.0	1.1	−2.0*	3.8	4.2
	8	RM3459-RM342	9	RM3164-RM6570	−1.3**	0.9	0.2	−1.1	3.2	2.5
SNC	12	RM6296-RM20	/	/	−5.1**	3.8**	4.2**	−8.2**	3.2	4.8
	5	RM267-RM7302	6	RM340-RM1370	1.6	−1.1	−5.3	6.5**	0.5	5.3
	8	RM223-RM3459	12	RM7344-RM1261	−6.6**	3.2	0.4	−3.7*	5.3	1.5
SKC	3	RM6832-RM7370	12	RM1337-RM101	0.3	−0.6	−2.0	2.5**	0.7	5.5
	3	RM6832-RM7370	12	RM7344-RM1261	0.2	−1.5	−1.6	3.1**	0.6	6.5
	5	RM1366-RM267	6	RM6811-RM340	2.0**	−2.2*	−3.0	5.0**	1.9	7.5
STR	6	RM6298-RM6818	12	RM5609-RM1103	−0.2**	/	0.0	0.0	4.6	0.7

a RNC: root Na^+^ concentration; RKC: root K^+^ concentration; SNC: shoot Na^+^ concentration; SKC: shoot K^+^ concentration; STR: salt tolerance rating;

b Chromosome on which the QTL was located;

c A or AA represents the estimated additive effect of additive QTL or epistatic QTL, and the AE1, AE2 and AE3 represent the additive effects of additive QTL under 0, 100 and 120 mM NaCl conditions, respectively; Its positive value indicates that Jiucaiqing has the positive allele and the case of negative values is just the opposite; AAE1, AAE2 and AAE3 represent the additive effects of epistatic QTL under 0, 100 and 120 mM NaCl conditions, respectively; Its positive value indicates that two loci genotypes being the same as those in parent Jiucaiqing (or IR26) take the positive effects, while the two-loci recombinants take the negative effects;

d r^2^(A), r^2^(AA), r^2^(AE) and r^2^(AAE) represent the phenotypic variation explained by the additive QTL, epistatic QTL, additive QTL× environment interactions and epistatic QTL×environment interactions, respectively.

** and * indicates significance at the level of 1% and 5%, respectively.

### Identification of Effects of QTL *qSNC11* on Seedling Salt Tolerance

**Figure 3:**
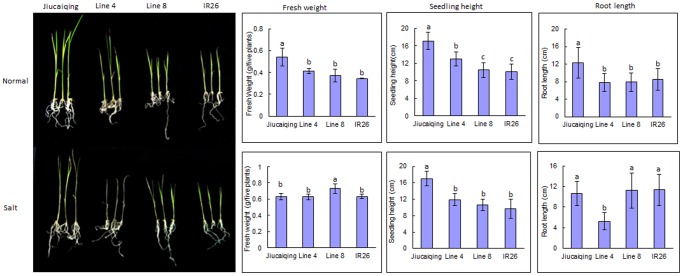
Effects of QTL *qSNC11* on seedling salt tolerance.

**Figure 4:**
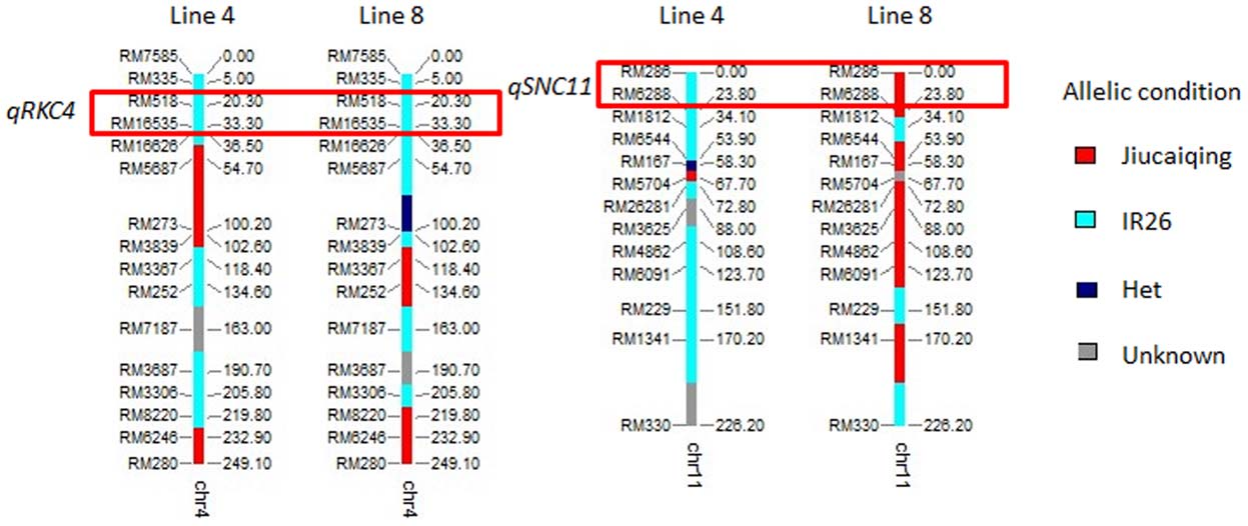
Graphical representation of the genotype of chromosomes located QTLs for lines 4 and 8.

Under 120 mM NaCl condition, two additive QTLs *qRKC4* and *qSNC11* were identified, and the positive alleles of both QTLs were contributed by Jiucaiqing variety to increase seedling salt tolerance. The rice seedling salt tolerance under 120 mM NaCl might be mainly affected by these two additive QTLs in this study. To identify the major effects of QTL *qSNC11*, the seedling salt tolerance of two lines (lines 4 and 8) were further evaluated under 120 mM NaCl (Fig. 3). The lines 4 and 8 have a relative high similar genotype (about 60% similarity), and the alleles of *qRKC4* in both lines were those of the IR26 parent, whereas the *qSNC11* allele of line 4 was from IR26 and that of line 8 was from Jiucaiqing (Fig. 4). The seedlings of line 8 survived and maintained a green leaf color, whereas the line 4 seedlings died (Fig. 3). There was no significant difference in the seedling fresh weight and root length between lines 4 and 8 under the control condition. Compared with line 4, the seedling fresh weight and root length of line 8 were significantly higher under the 120 mM NaCl condition. Taken together, these results indicate that *qSNC11* might play an important role in rice seedling salt tolerance under 120 mM NaCl stress.

## Discussion

Accurate phenotyping is important for QTL mapping. At the seedling stage, the capacity to maintain a low Na^+^ concentration or a high K^+^ in leaves or roots is considered an indicator of potential salt tolerance in rice [39]–[41]. In this study, the correlations between the STR and Na^+^ or K^+^ concentration under NaCl stress supported these results. The salt concentration for the treatment is particularly important for effective evaluation of rice salt tolerance. Therefore, it was appropriate to jointly estimate the rice seedling salt tolerance using the indices of STR and Na^+^ or K^+^ concentrations in leaves or roots under different salt concentrations.

Previous studies showed that the recirculation of Na^+^ from shoots to roots was important for detoxifying Na^+^ [6]. Our results suggested that the Na^+^ was increased more in shoots than in roots under 100 and 120 mM NaCl conditions, compared to the control condition. A similar result was observed for K^+^. The processes of Na^+^ and K^+^ uptake in rice were considered to be independent under salt stress because the major pathways of Na^+^ and K^+^ uptake in rice occur in parallel and not in direct competition [10], [42]–[43]. Similar results were observed in our study in that the significantly positive correlations between SNC and SKC in shoots and between RNC and RKC in roots were observed under salt stress.

The use of three different NaCl concentrations greatly facilitated the detection of QTLs, also allowing for the identification of QTL×environment interactions. As a result, nine and seven additive QTLs were identified by CIM and ICIM, respectively, and five of them were identified by both programs. These five additive QTLs may be considered as reliable QTLs. We found that the expressions of additive QTLs for Na^+^ and K^+^ under the control and salt stress conditions in rice were different, so that additive QTLs rarely co-localized among the different NaCl conditions. This finding suggests that additive QTLs are affected by environmental factors.

The QTLs detected in seedling shoots and roots were quite different. The QTLs for SNC and RNC and QTLs for SKC and RKC did not map to the same locations. Similarly, the QTLs detected for Na^+^ and K^+^ concentrations were also quite different. The QTLs for RNC and RKC did not map to the same locations, and the same map location was found only for the QTL for SNC and SKC (*qSNC* and *qSKC*). These results suggest that the loci controlling the transport of two ions, Na^+^ and K^+^, between shoots and roots of seedlings may be different or induced differentially by salt stress, similar to the results of Lin et al. [12].

With the integration of different linkage maps of RGP and the updating of Gramene database (http:// www.gramene.org/), it is possible to compare roughly the QTLs found in different research groups according to the physical location of markers linked with QTLs. In rice, a number of additive QTLs for salt tolerance have been identified. In our study, a total of eight additive QTLs were identified under salt stress condition. By comparing the chromosomal positions of these additive QTLs, several QTLs identified in previous studies were found to lie near the additive QTLs identified in this study. For example, we found that *qSNC11* was near the region of *QSkc11* and *QKna11* for rice seedling salt tolerance, and that *qSNC3* was located in a position that coincided with the region of a QTL (*QSst3b*) for score of leaf salt toxicity symptoms [16]. In addition, *qSKC4* mapped near the QTL *qRKC-4* for root K^+^ concentration in rice seedlings [12]. There were no QTLs previously reported to be close to *qSNC9*, *qSKC1*, *qSKC9*, *qRKC4* and *qSTR7*, which indicates that these additive QTLs might be novel salt tolerance loci. Further study is necessary to confirm these QTLs using near isogenic lines (NILs). With the increase of number of QTLs identified for salt tolerance, the genetic control of rice salt tolerance will be better understood, and a MAS strategy could become a promising approach for improving salt tolerance.

In general, if the additive QTL identified explains most of genotypic variation, i.e., the proportion of phenotypic variation explained is close to broad-sense heritability, epistasis is less important [28]. In this study, the total phenotypic variances explained by additive QTLs were relatively lower compared with the heritability of Na^+^ and K^+^ in shoots and roots under different salt stress conditions, indicating that there were epistatic QTLs or interactions between the QTLs and environment. Finally, one additive QTL and nine epistatic QTLs were identified by joint analyses, suggesting that epistatic QTLs and QTL×environment interactions are important components for Na^+^ and K^+^ concentrations in shoots and roots. For example, there were no additive QTLs for Na^+^ concentration in roots, while the Na^+^ concentration in roots was controlled by epistatic QTLs and QTL×environment interactions.

Several major QTLs for Na^+^ or K^+^ concentrations have been identified in different salt-tolerant rice varieties. For example, the salt-tolerant variety Pokkali was the source of positive alleles for the major QTL *Saltol* on chromosome 1 [11], accounting for high K^+^ and low Na^+^ absorptions and a low Na^+^/K^+^ ratio under salinity stress [44]. Lin et al. [12] identified a major QTL *qSKC-1* for the control of shoot K^+^ concentration in the same region of chromosome 1, and the allele of highly salt-tolerant variety Nona Bokra at this locus increased K^+^ concentration in shoot. Ren et al. [6] cloned the *SKC1* locus, and showed that the SKC1 protein is highly similar to HKT-type transporters. In this study, one novel major QTL *qSNC11* was identified with a relatively higher contribution to the variation and additive effects, and the allele of the salt-tolerant variety Jiucaiqing at this locus decreased Na^+^ concentration in shoots. Through the analysis of selected RILs with Jiucaiqing alleles at *qSNC11*, we found that the *qSNC11* locus was able to confer salt tolerance, thus providing an opportunity for marker-assisted backcrossing to improve the salt tolerance of commercial varieties.
